# Opposing Associations of Stress and Resilience With Functional Outcomes in Stroke Survivors in the Chronic Phase of Stroke: A Cross-Sectional Study

**DOI:** 10.3389/fneur.2020.00230

**Published:** 2020-04-22

**Authors:** Prajwal Gyawali, Wei Zhen Chow, Madeleine Hinwood, Murielle Kluge, Coralie English, Lin Kooi Ong, Michael Nilsson, Frederick Rohan Walker

**Affiliations:** ^1^School of Biomedical Sciences and Pharmacy and Priority Research Centre for Stroke and Brain Injury, The University of Newcastle, Callaghan, NSW, Australia; ^2^Hunter Medical Research Institute, New Lambton Heights, NSW, Australia; ^3^NHMRC Centre of Research Excellence in Stroke Rehabilitation and Brain Recovery, Heidelberg, VIC, Australia; ^4^School of Medicine and Public Health, University of Newcastle, Callaghan, NSW, Australia; ^5^Centre for Rehab Innovations, The University of Newcastle, Callaghan, NSW, Australia; ^6^School of Health Sciences, University of Newcastle, Callaghan, NSW, Australia; ^7^School of Pharmacy, Monash University Malaysia, Bandar Sunway, Malaysia

**Keywords:** stroke recovery, stress, resilience, mood, emotion, cognition

## Abstract

Stroke survivors report significant levels of psychological distress post stroke. To date, most studies conducted have focused on the relationship between psychological stress and functional outcomes in the acute phase of stroke. However, no studies had considered the role of stress over the chronic phase, where stress may continue to exert negative effects on cognitive and psychological processes. Further, the role of potentially modulatory variables, such as psychological resilience, on stroke outcomes has been understudied. The purpose of this study was to consider the relationships between stress and resilience with functional outcomes in long-term survivors of stroke. People (*N* = 70) who had experienced a stroke between 5 months and 28 years ago were included in the cross-sectional study, along with age-matched controls (*N* = 70). We measured stress using both the Perceived Stress Scale and biological markers, and resilience using both the Brief Resilience Scale and the Connor-Davidson Resilience Scale. Stroke outcomes were assessed using the Stroke Impact Scale. We found that, compared with age-matched controls, stroke survivors reported greater levels of perceived stress, and lower levels of resilience. In stroke survivors, both perceived stress and resilience were independently associated with stroke outcomes in linear regression models. In particular, these relationships were observed for cognitive outcomes including mood, memory, and communication. The association between stress and stroke outcome did not differ across time post stroke. Given that resilience is a modifiable psychological construct, future research may consider whether strategies directed at enhancing resilience may improve recovery from stroke.

Australia and New Zealand Clinical Trials Registry: ACTRN12617000736347.

## Introduction

Psychological stress refers to the negative emotional states generated when an individual perceives that they do not have the resources to cope with or respond to a threat, whether that threat is real or imagined (1). When stress is experienced repeatedly, or is severe and persistent in nature, it is almost always associated with negative health outcomes. For instance, chronic stress has been found to precede the development of depression, anxiety, diabetes, and cardiovascular disease, as well as contribute other poor health outcomes such as immunosuppression, fatigue, apathy, and emotional lability (2–4). Psychological stress is therefore a likely modulator of long-term cognitive changes associated with stroke.

To date the investigation of stress in stroke survivors has been relatively limited, with a particular emphasis given over to considering stress levels within ~12 months of infarction. Several studies have identified that greater levels of perceived stress in the hyper-acute (+72 h post-stroke) and acute (14 days post-stroke) periods post-stroke were associated with worse outcomes (5, 6). Over a longer time frame, Ostwald et al. identified a relationship between self-reported stress, using the ten-item Perceived Stress Scale (PSS-10), and functional outcomes at 3, 6, 9, and 12 months post-stroke (7). Each item on the PSS-10 is rated on a 5-point scale (0 = never to 4 = very often), with an overall range of 0–40, with a higher score signifying a higher level of stress. The authors noted that mean PSS scores were ~12 (SD ~7) at discharge and declined slightly across the first 12 months, and that function was a significant predictor of stress levels for stroke survivors. Similarly, Dos Santos et al., followed 56 stroke survivors for 6 months following discharge, and observed a strong relationship between the levels of functional independence and perceived stress (8). A recent systematic review of 48 studies showed that elevated cortisol, a stress biomarker, is associated with increased dependency, morbidity, and mortality post-stroke (9). Although these studies have examined relationships between stress and broad functional stroke outcomes for up to 24 months post-stroke, increasing survival rates mean that stroke survivors may live for several decades following stroke onset. Knowledge around the impact of stress over these longer time frames remains limited.

The impact of stress post-stroke on cognitive and psychological outcomes in particular has been less well-characterized. Recently, however, the published results of the Tel Aviv Brain Acute Stroke Cohort (TABASCO) study examining a number of predictors for post-stroke outcomes are notable. In a prospective characterization of 182 stroke survivors, the study authors observed that levels of bedtime salivary cortisol levels immediately post-stroke (*N* = 182), and hair cortisol levels (used as an index of persistent stress) at 6, 12, and 24 months post-stroke (*N* = 65), were associated with significantly poorer cognitive function at these same time points (10, 11).

Collectively, those studies that have examined the impact of stress on outcomes post-stroke suggest that perceived stress and stress biomarkers predict worse cognitive, functional, and dependency status. There are, however, several components of the relationship between stress and cognition that have yet to be characterized, in particular the influence of resilience, a well-recognized modulator of stress. Apart from having purely theoretical interest, the relationship between stress, resilience, and stroke outcomes is a salient as there are several well characterized resilience building strategies available that could be deployed to modulate the negative effect of stress on outcomes (12).

Resilience is often defined as the ability to “bounce back” after experiencing a stressful or otherwise challenging event (13), or to adapt quickly and effectively to stress (14). There is a strong delineation between the common cognitive changes after stroke and the skills required for resilience. For example, increased rates of emotional lability, anxiety, depression, and poor communication skills are all common outcomes post-stroke (15–17). In contrast, traits such as emotional stability, optimism, self-regulation, problem solving skills, and effective communication are associated with resilience (12, 18). Resilience and changes in resilience post-stroke may explain variability in cognitive symptoms post-stroke.

The inverse relationship between stress and resilience has led to the hypothesis that the qualities that contribute to resilience may be capable of limiting the intensity of stress and in doing so mitigate many of the associated negative health outcomes (18). Resilience and vulnerability to stress is one of the most important topics in the field of stress research, and offers a potential point of intervention that will improve the rehabilitation of individuals after stroke (19).

The overall aim of this study was to examine stress levels in community-dwelling stroke survivors in the chronic phase of recovery from stroke, and consider the potential relationship between stress, resilience and a number of stroke outcomes during this period. Specifically, it was hypothesized that stroke survivors in the chronic phase of stroke will have higher levels of perceived stress and stress biomarkers than age matched controls; that higher stress levels will be associated with worse cognitive and emotional outcomes on the Stroke Impact Scale (SIS); and that greater levels of resilience will be associated with better cognitive and emotional outcomes on the SIS. We also explore whether the relationship between stress and cognitive or emotional outcomes is affected by time post stroke. This cross-sectional study was reported in accordance with the STROBE guidelines for reporting observational studies (20).

## Materials and Methods

This study reports the predefined primary objective of the cross-sectional case-control “Stress in people recovering from stroke” study registered in the Australian and New Zealand Clinical Trials Register (ACTRN12617000736347).

### Participants

Participants (*N* = 140) were recruited between November 2017 and February 2019. Community-dwelling stroke survivors in the chronic phase of stroke recovery (≥5 months post-stroke) were recruited via the Hunter Stroke Research Volunteer Register based at the Hunter Medical Research Institute (HMRI). The Hunter Stroke Research Volunteer Register includes over 600 stroke survivors, primarily residing in the Hunter New-England Health region of New South Wales, Australia. People who met the eligibility criteria were contacted via email or phone and informed about the study. Those who were interested were fully informed about the study, and provided with a participant information sheet. As part of the recruitment process, stroke survivors and/or their families or carers were given reassurance that there was no obligation for them to participate in this study. Stroke survivors who provided informed consent visited the study site either independently or with assistance from community workers or family members. Control participants were recruited either from the HMRI control registry, which is a register of people interested in participating in research projects run through HMRI, or via social media advertisements. Control participants were age-matched to stroke survivors, and had no history of stroke. Ethical approval for this study was obtained from the Hunter New England Local Health District Human Research Ethics Committee (17/06/21/4.02). Written informed consent was obtained from all participants before the study. Exclusion criteria included a history of pituitary and adrenal gland diseases. Our sample comprised 70 stroke survivors with a mean age of 62 years, and 70 age-matched controls with a mean age of 65 years.

### Assessments

All data were collected by 2 researchers who received training to reliably complete all assessments. Participants completed all experimental procedures at a single study visit, which lasted ~2 h. Data were collected on demographic characteristics (age, sex), anthropometrics (height, weight, waist circumference, and blood pressure), self-reported clinical history of comorbid conditions (participants asked to indicate whether they had a previous history of clinically diagnosed diabetes mellitus, dyslipidemia, mental illness, or hypertension), and self-reported level of physical activity. We used a single-item question: “How many times per week do you engage in intense physical activity—enough to work up a sweat,” as a self-reported measure of physical activity. This question has been validated (21), and has previously been used in a large longitudinal study to assess physical activity (22). Self-report type and date of last stroke was collected from stroke survivors. Waist circumference of the participants was measured from the upper margin of the posterior iliac crest at the end of normal expiration. Brachial blood pressure was measured twice with an automated blood pressure machine in a sitting position.

### Variables

All measures used Likert scales to obtain responses. In order to include participants with aphasia, participants were presented with paper versions of each scale, and each response item was also posed verbally. Participants could then indicate either verbally or by pointing where their response lay on the scale. All responses were self-reported for both stroke survivor and control participants.

Stress was measured using both biological markers, and self-report (10-item Perceived Stress Scale). Stress biomarkers in serum, including blood cortisol and co-peptin were collected from peripheral venous blood samples. About 10 mLs of blood was collected from all participants in EDTA and plain vials. The blood sample was centrifuged immediately and aliquots of plasma and serum samples were stored in Eppendorf tubes at −80°C. Samples were processed using commercially available ELISA kits as per the manufacturer's instructions (Cortisol: Stratech Scientific APAC Pty Ltd; Co-peptin: CUSABIO.

The Perceived Stress Scale (PSS-10), a 10-item scale, was used to measure perceived psychological stress in all participants asking them to rate how stressful they perceived their life to be during the previous month (23). Item scores were rated on a 5-point scale (0 = never to 4 = very often), and the overall score ranges from 0 to 40, with higher scores suggesting higher levels of stress. The PSS-10 has been widely shown to have acceptable psychometric properties (24), and has been used previously in stroke survivors (8).

The Brief Resiliency Scale (BRS) (13) and the Connor-Davidson Resilience Scale (14) were used to measure resilience in participants. Resilience was measured using two different self-report measures, each thought to explore different aspects of the construct. The Brief Resilience Scale (BRS) assesses successful recovery from stressful experiences (13). The Connor-Davidson Resilience Scale (CD-RISC) is considered to assess an alternative definition of resilience; that is the concept of thriving or maintaining a stable trajectory of mental health throughout a period of adversity (14). Interestingly, several studies have shown that the BRS and CD-RISC share only a moderate cross-correlation indicating that they likely capture different phenomena (13, 24, 25).

The BRS is a six-item scale (0 lowest and 5 highest resilience score), aimed at assessing trait resilience; that is, the ability to recover from stress. The BRS was used to measure resilience in both stroke survivors and control participants. The BRS has been previously shown to be a valid and reliable means to assess resilience as the ability to “bounce back” (25). The BRS has previously been used in intervention studies conducted in stroke survivors (19).

The Connor-Davidson Resilience Scale (CD-RISC) was used to measure resilience in stroke survivors only. This scale is thought to assess the ability to effectively adapt to and withstand stressful conditions through the application of certain resources, referred to as thriving despite adversity (25). The CD-RISC comprises 25 statements on how one has felt over the past month. The response scale has a 5-point range, and the possible overall score ranges from 0 (low resilience) to 100 (high resilience). The CD-RISC has been shown to be reliable and valid in a number of populations, and has been previously used in stroke survivors (26).

Stroke outcome was measured using the Stroke Impact Scale (SIS) Version 3.0 (27). The SIS is a well-validated stroke specific, quality of life scale that assesses the degree to which the physical, mental, and emotional changes due to stroke affect the survivor's quality of life (28, 29). It includes 64 items across 8 domains: physical problems, memory and thinking, mood and emotion, communication, activities of daily living, mobility, hand function, and participation and role function, thus representing the comprehensive health measures across the full impairment-participation continuum. Each item is measured using a 5-point Likert scale, and the possible standardized score for each domain ranges from 0 to 100. An additional question on stroke recovery asks that the stroke survivors rate on a scale from 0 to 100 how much they feel that they have recovered from their stroke overall, with 0 being no recovery to 100 being fully recovered. The SIS demonstrates good psychometric properties, including internal consistency, reliability, and validity, and is considered to be clinically relevant and a good measure of quality of life (28, 29).

### Sample Size

Sample size was calculated for the comparison of perceived and biological measures of stress between stroke survivors and control, based on the previous estimates of population variability (mean differences and SD) for the primary outcome measures (PSS-10, and cortisol) for a two-tailed test testing using alpha = 0.05 and beta = 0.2. The maximum required sample size was calculated to be 60 in each group; 70 participants were recruited per group to allow for attrition.

### Co-variables

In this study, the independent variables were measures of stress and resilience, and the outcome variable was stroke outcome. Analyses were adjusted for relevant co-variables identified using evidence from previous literature. In the comparisons between stroke survivors and controls, analyses were adjusted for age, sex, level of physical activity, history of mental illness, and dyslipidaemia. Multivariate linear regression analyses examining the contributions of stress and resilience to stroke outcomes were adjusted for the following confounding variables: age, sex, stroke type, time since stroke, self-reported history of mental illness, and level of physical activity.

### Statistical Analysis

Analyses were conducted using SPSS version 25.0 software (IBM Corp, 2017). Pearson correlations were used to examine crude associations between stress, resilience, and stroke outcomes (all domains of the Stroke Impact Scale) in stroke survivors. Linear regression analysis was used to compare measures of stress and resilience (BRS) between stroke survivors and age-matched controls, and to compare differences in the level of perceived stress over time post stroke. Independent variables (perceived stress and resilience) were mean-centered. Multivariate linear regression was used to examine of the association between stress and resilience and stroke outcomes. Where a significant proportion of values are reported as missing, sensitivity analysis using multiple imputation was used to test the impact of these missing values. Multiple imputation was performed using the default option in SPSS. Briefly, five datasets were generated with the missing values imputed at random. The imputed datasets were then pooled, and the average used in sensitivity analysis. Unadjusted models are presented in the Supplementary Appendix.

Significance was set at *p* < 0.05 (two tailed), with a Bonferroni correction applied for multiple comparisons in the linear regression analyses conducted in the stroke survivor group (27 analyses: *p* = 0.05/27). Crude (unadjusted) models are presented in the online Supplementary Appendix.

## Results

### Baseline Demographics

A total of 70 stroke survivors ranging from 5 months to 28 years post-stroke (median 38.5 months), and 70 age-matched controls participated in the study. Table 1 presents demographic characteristics for both stroke survivors and age-matched control participants, and stroke outcomes for stroke survivors, with comparisons performed via *t*-test (continuous variables) or Chi-square tests (categorical variables).

**Table 1:** Comparison of demographic and clinical data.

	**Stroke survivors *N* = 70**	**Controls *N* = 70**	***P***
**DEMOGRAPHIC CHARACTERISTICS**			
Age, mean years (SD)	61.9 (13.8)	64.6 (10.0)	0.192
Gender, male *N* (%)	38 (54.3)	24 (34.3)	0.027
BMI, mean kg/m^2^ (SD)	29.01 (6.3)	28.0 (5.7)	0.332
Waist circumference, mean cm (SD)	98.7 (21.5)	95.4 (15.5)	0.301
**CLINICAL CHARACTERISTICS**			
Systolic blood pressure, mean mmHg (SD)	131 (17)	131 (18)	0.985
Diastolic blood pressure, mean mmHg (SD)	78 (12)	79 (6)	0.724
Physical activity, mean sessions per week (SD)	1.0 (0.9)	1.2 (0.7)	0.222
Self-reported history of:			
- Diabetes, *n* (%)	10 (14.3)	6 (8.6)	0.234
- Hypertension, *n* (%)	28 (40.0)	21 (30.0)	0.131
- Dyslipidaemia, *n* (%)	38 (54.3)	16 (22.9)	<0.001
- Mental illness, *n* (%)	15 (21.4)	11 (15.7)	0.302
**STROKE CHARACTERISTICS**			
Stroke type (I/H/unknown)	41/26/3		
Time since stroke, median months (IQR)	38.5 (13.75, 117.50)		
Standarised Stroke Impact Scale items, mean score (SD):			
SIS 1: Physical problems	62.9 (26.7)		
SIS 2: Memory and thinking	71.33 (21.3)		
SIS 3: Mood and emotion	73.8 (17.0)		
SIS 4: Communication	78.9 (19.9)		
SIS 5: Activities of daily living	80.1 (21.0)		
SIS 6: Mobility	78.1 (20.8)		
SIS 7: Hand function	60.1 (36.4)		
SIS 8: Participation/role function	63.9 (23.9)		
SIS 9: Overall perception of recovery	67.8 (18. 8)		

Overall, demographic characteristics of stroke survivors and matched controls were similar, except for gender distribution and dyslipidaemia.

For analysis the SIS index was calculated by summing scores of the 8 items and then standardizing each score on a scale of 0 to 100.

### Perceived Stress and Resilience Differs in Stroke Survivors Compared With Controls

Table 2 presents results for perceived stress, resilience, and stress biomarkers for stroke survivors and control participants, with comparisons performed via linear regression analysis.

**Table 2:** Comparison of stress and resilience measures between controls and stroke survivors.

	**Controls, mean (SE)**	***n***	**Stroke survivors, mean (SE)**	***n***	**Adjusted^a^ B (95% CI)**	***p***
PSS-10	11.43 (0.7)	70	16.90 (0.8)	70	6.12 (8.62, 3.73)	<0.001
Serum cortisol (μg/dL)	9.3 (0.4)	70	7.8 (0.4)	68	−1.83 (−0.51, −3.15)	0.007
Copeptin (pg/mL)	174.6 (11.9)	70	163.8 (14.1)	68	−9.01 (−50.49, 32.47)	0.668
BRS	4.0 (0.1)	69	3.5 (0.1)	70	−0.48 (−0.14, −0.82)	0.006
CD-RISC	NR	–	69.1 (2.2)	66	–	–
Cort 1st segment (pg/mg)	14.4 (1.8)	59	14.8 (2.6)	60	2.37 (−4.87, 9.86)	0.518
Cort 2nd segment (pg/mg)	16.2 (2.2)	55	14.4 (2.4)	50	3.10 (−4.16, 10.37)	0.398
**Sensitivity analysis: Hair cortisol with multiple imputation for missing at random**						
Cort 1st segment (pooled result)					1.79	0.61
Cort 2nd segment (pooled result)					3.04	0.35

a *Adjusted for age, sex, dyslipidemia, level of physical activity, history of mental illness*.

*BRS, brief resilience Scale; CD-RISC, Connor-Davidson Resilience Scale; CI, confidence interval; PSS-10, 10 item Perceived Stress Scale; SE, standard error mean; NR, not recorded*.

Stroke survivors reported statistically significantly greater perceived stress than age-matched controls. Serum cortisol was statistically significantly lower in stroke survivors than in control participants. Stroke survivors reported statistically significantly lower resilience (BRS) than controls. There was no statistically significant difference between stroke survivors and controls for serum copeptin, and hair cortisol (first and second segments). Due to a large fraction of missing values for hair cortisol, multiple imputation for missing values (5 imputations) was performed and the pooled result reported, which was consistent with the base case analysis.

### Perceived Stress and Resilience Are Both Associated With Stroke Outcomes

Table 3 summarizes Pearson correlations between the study variables, in order to analyse crude bivariate relationships between stroke outcome, resilience, and perceived stress for stroke survivors.

**Table 3:** Pearson correlations between stress, resilience, and stroke outcome measures (stroke survivors).

	**PSS-10**	**CD-RISC**	**BRS**
PSS-10	1		
CD-RISC	−0.451^**^	1	
BRS	−0.653^**^	0.585^**^	1
SIS1	−0.256^*^	0.164	0.280^*^
SIS2	−0.459^**^	0.371^**^	0.402^**^
SIS3	−0.580^**^	0.432^**^	0.511^**^
SIS4	−0.405^**^	0.319^**^	0.536^**^
SIS5	−0.332^**^	0.239	0.259^*^
SIS6	−0.296^*^	0.157	0.150
SIS7	−0.197	0.087	0.127
SIS8	−0.465^**^	0.340^**^	0.444^**^
SIS9	−0.399^**^	0.337^**^	0.456^**^

** *Correlation is significant at the 0.01 level (2-tailed)*.

* *Correlation is significant at the 0.05 level (2-tailed)*.

*BRS, Brief Resilience Scale; CD-RISC, Connor-Davidson Resilience Scale; PSS-10, 10-item Perceived Stress Scale; SIS, Stroke Impact Scale*.

*Stroke Impact Scale items: 1, Physical problems; 2, Memory and thinking; 3, Mood and emotion; 4, Communication; 5, Activities of daily living; 6, Mobility; 7, Hand function; 8, Participation/role function; 9, Overall perception of recovery*.

Perceived stress was moderately negatively correlated with resilience (both CD-RISC and BRS), and more weakly negatively correlated with most items (excepting Item 7: Hand function) on the SIS (all *p* < 0.05). Resilience as measured by the CD-RISC was moderately correlated with resilience measured using the BRS. Resilience (CD-RISC) was weakly-moderately positively correlated with a number of outcome measures on the SIS (Memory and thinking; Mood and emotion; Communication; Participation/role function; and Overall perception of recovery; all *p* < 0.05). Resilience (BRS) was also weakly-moderately positively correlated with a number of outcome measures on the SIS (Physical problems; Memory and thinking; Mood and emotion; Communication; Activities of daily living; Participation/role function; and Overall perception of recovery; all *p* < 0.05). Neither measure of resilience was correlated with SIS items 6 or 7 (Mobility and Hand function). Stress and resilience were also only weakly correlated with SIS item 1 (Physical Problems).

Multivariate linear regression models examining the association between perceived stress and stroke outcomes are summarized in Table 4.

**Table 4:** Main effect of perceived stress (PSS-10) on Stroke Impact Scale (SIS).

**Outcome**	**Exposure**	**Unstandardized coefficients**		**standardized coefficients**	**T**	**Adj. *p***	***R*^**2**^**	**F**
		**Beta (95% CI)**	**SE**	**Beta**				
Physical problems							0.213	2.163
	Intercept	60.11 (9.17, 11.04)	25.43		2.36	0.022		
	PSS-10	−0.86 (−1.85, 0.14)	0.50	−0.22	−1.73	0.089		
Memory and thinking							0.287	3.227
	Intercept	87.53 (51.20, 123.87)	18.14		4.83	<0.001		
	PSS-10^*^	−1.30 (−2.01, −0.59)	0.35	−0.44	−3.66	<0.001		
Mood and emotion							0.353	4.366
	Intercept	64.79 (35.95, 93.63)	14.40		4.50	<0.001		
	PSS-10^*^	−1.28 (−1.84, −0.72)	0.28	−0.52	−4.55	<0.001		
Communication							0.277	3.062
	Intercept	66.55 (31.4, 101.69)	17.54		3.79	<0.001		
	PSS-10	−1.10 (−1.78, −0.41)	0.34	−0.39	−3.21	0.002		
Activities of daily living							0.254	2.731
	Intercept	93.62 (55.63, 131.60)	18.96		4.94	<0.001		
	PSS-10	−0.80 (−1.54, −0.06)	0.37	−0.27	−2.15	0.036		
Mobility							0.295	3.350
	Intercept	83.32 (45.94, 120.71)	18.66		4.47	<0.001		
	PSS-10	−0.82 (−1.55, −0.09)	0.36	−0.27	−2.25	0.029		
Hand function							0.133	1.227
	Intercept	63.27 (−9.19, 135.73)	36.17		1.75	0.086		
	PSS-10	−1.05 (−2.46, 0.37)	0.71	−0.20	−1.48	0.144		
Participation/role function							0.254	2.731
	Intercept	53.58 (9.81, 97.36)	21.85		2.45	0.017		
	PSS-10^*^	−1.58 (−2.43, −0.26)	−0.43	−0.46	−3.70	<0.001		
Overall perception of recovery							0.240	2.524
	Intercept	69.85 (35.53, 104.17)	17.13		4.08	<0.001		
	PSS-10	−0.93 (−1.60, −0.52)	0.33	−0.34	−2.77	0.008		

*Adjusted for age, sex, stroke type, time since stroke, physical activity, history of mental illness*.

*PSS-10 mean centered. Threshold p-value 0.00185 to account for multiple comparisons*.

* *Statistically significant*.

Perceived stress was statistically significantly associated with 3 of the 9 stroke impact scale items (Memory and Thinking; Mood and Emotion; and Participation/Role Function) in the respective linear regression models, after adjusting for age, sex, stroke type, time since stroke, self-reported history of mental illness, and physical activity. Higher levels of perceived stress were associated with poorer scores on the Stroke Impact Scale.

Multivariate linear regression models examining the association between resilience, as measured using BRS, and stroke outcomes are summarized in Table 5.

**Table 5:** Main effect of resilience (BRS) on Stroke Impact Scale (SIS).

**Outcome**	**Exposure**	**Unstandardized coefficients**		**standardized coefficients**	**T**	**Adj. *p***	***R*^**2**^**	**F**
		**Beta (95% CI)**	**SE**	**Beta**				
Physical problems							0.238	2.497
	Intercept	63.32 (13.36, 113.27)	24.94		2.54	0.014		
	BRS	9.29 (0.91, 17.68)	4.19	0.31	2.22	0.03		
Memory and thinking							0.209	2.110
	Intercept	92.61 (54.45, 130.77)	19.05		4.86	<0.001		
	BRS	8.16 (1.76, 14.56)	3.20	0.36	2.55	0.013		
Mood and emotion							0.272	2.983
	Intercept	69.77 (39.27, 100.27)	15.23		4.58	<0.001		
	BRS^*^	8.91 (3.79, 14.03)	2.56	0.47	3.49	<0.001		
Communication							0.360	4.505
	Intercept	70.65 (37.71, 103.60)	16.45		4.30	<0.001		
	BRS^*^	12.01 (6.48, 17.54)	2.76	0.55	4.35	<0.001		
Activities of daily living							0.241	2.538
	Intercept	96.70 (58.49, 134.90)	19.07		5.07	<0.001		
	BRS	6.03 (−0.39, 12.44)	3.20	0.26	1.88	0.065		
Mobility							0.248	2.637
	Intercept	86.59 (48.10, 125.08)	19.21		4.51	<0.001		
	BRS	3.56 (−2.90, 10.02)	3.22	0.15	1.10	0.274		
Hand function							0.128	1.172
	Intercept	67.30 (−5.14, 139.74)	36.16		1.86	0.068		
	BRS	8.24 (−3.92, 20.40)	6.07	0.20	1.36	0.180		
Participation/role function							0.233	2.424
	Intercept	59.65 (15.38, 103.92)	22.10		2.70	0.009		
	BRS^*^	12.67 (5.24, 20.10)	3.71	0.47	3.42	<0.001		
Overall perception of recovery							0.303	3.473
	Intercept	73.32 (40.56, 106.08)	16.35		4.48	<0.001		
	BRS^*^	10.06 (4.56, 15.55)	2.74	0.48	3.66	<0.001		

*Adjusted for age, sex, stroke type, time since stroke, physical activity, history of mental illness*.

*BRS mean centered. Threshold p-value 0.00185 to account for multiple comparisons*.

* *Statistically significant*.

Resilience (as measured using the BRS) was statistically significantly associated with 4 of the 9 Stroke Impact Scale items (Mood and Emotion; Communication; Participation/Role function; and Overall perception of recovery), in the respective linear regression models after adjusting for age, sex, stroke type, time since stroke, self-reported history of mental illness, and physical activity. A higher score on the BRS was associated with an improved outcome on the SIS scales.

Multivariate linear regression models examining associations between resilience, as measured using the CD-RISC, and stroke outcomes are summarized in Table 6.

**Table 6:** Main effect of resilience (CD-RISC) on Stroke Impact Scale (SIS).

**Outcome**	**Exposure**	**Unstandardized coefficients**		**standardized coefficients**	**T**	**Adj. *p***	***R*^**2**^**	**F**
		**Beta (95% CI)**	**SE**	**Beta**				
Physical problems							0.203	2.006
	Intercept	64.24 (12.29, 116.20)	25.92		2.48	0.0165		
	CD-RISC	0.31 (−0.09, 0.71)	0.20	0.20	1.55	0.128		
Memory and thinking							0.224	2.268
	Intercept	89.47 (51.62, 127.32)	18.89		4.74	<0.001		
	CD-RISC	0.42 (0.13, 0.71)	0.15	0.37	2.87	0.006		
Mood and emotion							0.219	2.209
	Intercept	71.51 (39.55, 103.47)	15.95		4.48	<0.001		
	CD-RISC	0.34 (0.10, 0.59)	0.12	0.36	2.78	0.007		
Communication							0.220	2.211
	Intercept	72.13 (35.19, 109.06)	18.43		3.91	<0.001		
	CD-RISC	0.34 (0.05, 0.62)	0.14	0.31	2.35	0.022		
Activities of daily living							0.211	2.106
	Intercept	92.66 (54.52, 130.80)	19.03		4.87	<0.001		
	CD-RISC	0.27 (−0.02, 0.57)	0.15	0.24	1.84	0.071		
Mobility							0.234	2.404
	Intercept	82.61(44.19, 121.03)	19.18		4.31	<0.001		
	CD-RISC	00.26 (−0.03, 0.55)	0.15	0.23	1.78	0.081		
Hand function							0.114	1.103
	Intercept	68.29 (−6.05, 142.62)	37.09		1.84	0.071		
	CD-RISC	0.28 (−0.29, 0.86)	0.29	0.14	0.99	0.328		
Participation/role function							0.170	1.604
	Intercept	60.29 (13.38, 107.21)	23.41		2.58	0.013		
	CD-RISC	0.46 (0.10, 0.82)	0.18	0.34	2.53	0.014		
Overall perception of recovery							0.240	2.486
	Intercept	71.91 (37.34, 106.48)	17.25		4.17	<0.001		
	CD-RISC	0.38 (0.11, 0.65)	0.13	0.37	2.85	0.006		

*Adjusted for age, sex, stroke type, time since stroke, physical activity, history of mental illness*.

*CD-RISC mean centered. Threshold p-value 0.00185 to account for multiple comparisons*.

*Statistically significant.

Resilience score (CD-RISC) was not statistically significantly associated with any of the Stroke Impact Scale items in the respective linear regression models after adjusting for age, sex, stroke type, time since stroke, history of mental illness, and physical activity.

### In Stroke Survivors, Perceived Stress Scores Are Similar Regardless of Time Post Stroke

**Figure 1:**
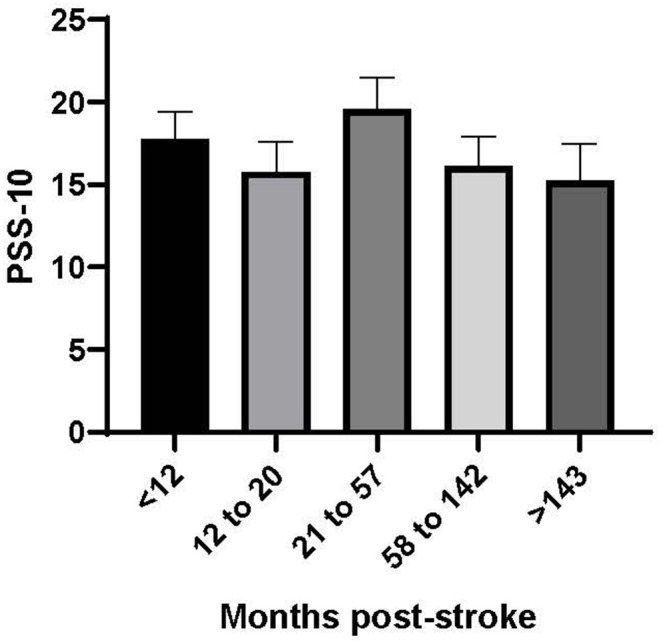
Perceived stress score (PSS-10) by time post-stroke (months). Time post-stroke is reported in quintiles (*n* = 14 per group).

There was significant heterogeneity in our dataset, with stroke survivors reporting being between 5 months and 28 years post-stroke (median 38.5 months). We therefore considered it prudent to investigate the relationship between stress and stroke outcomes as a function of time post stroke. Figure 1 shows the mean (SE) perceived stress scores over time post stroke.

Figure 1 shows that stroke survivors reported a consistent level of perceived stress regardless of time post stroke. A linear regression model examining the relationship between time post stroke and perceived stress score was not statistically significant (*p* = 0.426).

Unadjusted multivariate linear regression models examining the interaction between perceived stress and time post-stroke on stroke outcomes are presented in Table 7 below.

**Table 7:** Multivariable linear regression analysis of interaction between time post stroke and perceived stress (PSS-10) on SIS outcomes.

		**Unstandardized coefficients**	**standardized coefficients**		**T**	**Adj. *p***	***R*^**2**^**	**F**
		**Beta (95% CI)**	**SE**	**Beta**				
Physical problems							0.075	1.780
	Intercept	65.02 (55.60, 74.4)	4.72		13.78	<0.001		
	PSS-10	−0.63 (−1.89, 0.64)	0.63	−0.17	−0.99	0.33		
	Time	0.00 (−0.08, 0.08)	0.04	0.01	0.06	0.96		
	Interaction	−0.00 (−0.01, 0.01)	0.01	−0.13	−0.80	0.43		
Memory and thinking							0.226	6.424
	Intercept	77.18 (70.33, 84.03)	3.43		22.49	<0.001		
	PSS-10	−1.74 (−2.66, −0.82)	0.46	−0.58	−3.78	<0.001		
	Time	−0.02 (−0.08, 0.04)	0.03	−0.08	−0.73	0.47		
	Interaction	0.00 (−0.00, 0.01)	0.00	0.16	1.04	0.30		
Mood and emotion							0.344	11.542
	Intercept	76.12 (71.09, 81.15)	2.52		30.21	<0.001		
	PSS-10	−1.25 (−1.92, −0.57)	0.34	−0.52	−3.69	<0.001		
	Time	0.02 (−0.03, 0.06)	0.02	0.08	0.81	0.42		
	Interaction	−0.00 (−0.01, 0.00)	0.00	−0.08	−0.54	0.59		
Communication							0.203	5.592
	Intercept	85.78 (79.25, 92.30)	3.27		26.25	<0.001		
	PSS-10	−1.23 (−2.10, −0.35)	0.44	−0.43	−2.80	0.01		
	Time	−0.05 (−0.10, 0.01)	0.03	−0.20	−1.73	0.09		
	Interaction	0.00 (−0.01, 0.01)	0.00	0.01	0.04	0.97		
Activities of daily living							0.178	4.756
	Intercept	80.45 (73.47, 87.42)	3.49		23.03	<0.001		
	PSS-10	−1.43 (−2.37, −0.49)	0.47	−0.48	−3.05	<0.01		
	Time	0.04 (−0.02, 0.10)	0.03	0.15	1.27	0.21		
	Interaction	0.01 (−0.00, 0.01)	0.00	0.25	1.56	0.12		
Mobility							0.110	2.728
	Intercept	78.89 (71.70, 86.08)	3.60		21.91	<0.001		
	PSS-10	−1.09 (−2.05, −0.12)	0.48	−0.37	−2.25	0.03		
	Time	0.03 (−0.04, 0.09)	0.03	0.10	0.81	0.42		
	Interaction	0.00 (−0.01, 0.01)	0.00	0.13	0.77	0.45		
Hand function							0.053	1.232
	Intercept	63.44 (50.48, 76.41)	6.49		9.77	<0.001		
	PSS-10	−0.53 (−2.27, 1.21)	0.87	−0.10	−0.61	0.55		
	Time	−0.02 (−0.13, 0.10)	0.06	−0.04	−0.28	0.78		
	Interaction	−0.01 (−0.02, 0.01)	0.01	−0.15	−0.86	0.39		
Participation/role function							0.219	6.162
	Intercept	67.30 (59.56, 75.04)	3.88		17.36	<0.001		
	PSS-10	−1.61 (−2.65, −0.57)	0.52	−0.47	−3.09	<0.01		
	Time	0.01 (−0.05, 0.08)	0.03	0.04	0.38	0.71		
	Interaction	0.00 (−0.01, 0.01)	0.00	0.02	0.14	0.89		
Overall perception of recovery							0.162	4.260
	Intercept	71.75 (65.45, 78.05)	3.15		22.75	<0.001		
	PSS-10	−1.11(−1.96, −0.27)	0.42	−0.42	−2.63	0.01		
	Time	−0.01 (−0.07, 0.04)	0.03	−0.06	−0.49	0.62		
	Interaction	0.00 (−0.01, 0.01)	0.00	0.02	0.12	0.91		

*Independent variable (PSS-10) is mean-centered*.

*PSS, Perceived Stress Scale-10; Time, time post-stroke*.

The linear regression models evaluating the effect of PSS-10 on the Stroke Impact Scale outcomes were not statistically significant, suggesting that the relationship between perceived stress and stroke outcomes does not appear to vary with length of time post stroke.

## Discussion

This cross-sectional study, which considered how stress and resilience relate to functional outcomes in stroke survivors, yielded several notable findings. First, we identified that stroke survivors in the chronic phase of stroke recovery reported higher levels of perceived stress, and lower levels of resilience, than age-matched controls. Secondly, we observed that perceived stress was negatively associated with stroke outcomes across cognitive and emotional domains, and that this relationship existed regardless of time post-stroke. Thirdly, we found that resilience was positively associated with stroke outcomes. As stress is recognized to be a major determinant in the subsequent emergence of psychopathology, especially depression (30), as well as accelerate neurodegenerative sequelae triggered by stroke, our findings suggest that the constructs of stress and resilience warrant further investigation for their potential role in mediating the long-term outcomes of stroke.

Our study population included stroke survivors in what may be referred to as the chronic phase of recovery, i.e., >5 months after the stroke event. In our sample, stroke survivors reported a median time since stroke of 38.5 months (~3 years), and nearly all patients were living in the community at the time of recruitment. This cohort demonstrates the heterogeneity inherent in stroke survivors. For comparison, we also considered stress and resilience levels in an age-matched control group. Four metrics were used to assess stress: self-reported stress (Perceived Stress Scale-10) (31, 32), serum cortisol, serum co-peptin, and hair cortisol, a measure of cortisol accumulation over time.

We observed that stroke survivors exhibited statistically higher levels of self-reported stress (mean = 16.9 [±0.8 SE]) on the PSS compared to those observed in age matched controls (mean = 11.4 [±0.7 SE]). These findings are aligned with previous work demonstrating that a significant burden of perceived stress exists in stroke survivors relative to matched controls (7, 8, 10). We also identified that perceived stress in stroke survivors appears to remain elevated relative to controls regardless of the length of time since stroke.

While we observed a clear difference amongst survivors in their self-reported levels of stress, we found no evidence of elevated levels of serum cortisol, hair cortisol, or co-peptin in stroke survivors relative to age matched controls. Given our PSS-10 results and earlier findings from the TABASCO study (10), we were surprised not to observe elevated levels of these stress biomarkers. One obvious difference between our study and the TABASCO study is that we had adopted a cross-sectional design, whereas the TABASCO study had adopted a within subject longitudinal design. If there is a substantial variance in the stress biomarkers relative to self-report measures than population heterogeneity may have diluted any potential effects. Further, several studies have documented significant circadian and ultradian rhythms in cortisol release (33, 34). One recent study, which measured saliva cortisol at bedtime and post-awakening on admission, and 6, 12, and 24 months after stroke, found that only bedtime cortisol correlated with cognitive outcomes post-stroke, suggesting that timing of measurement is important (11). A within subjects longitudinal design can mitigate against greater inherent variance in the dependent variable. Ideally, we could have used such a design, however this was not an option as the recruitment of patients in our region would have inflated the study time window. Clearly, a priority of future work is to increase the number of participating sites to allow a larger within-subjects cohort to be recruited.

We also observed that stroke survivors reported lower levels of resilience (mean = 3.5 [±0.1 SE]) compared with matched controls (mean = 4.0 [±0.1 SE]) using the Brief Resilience Scale (BRS). In a recent review of resilience measures, the BRS was identified as being unique in assessing resilience as an outcome (the ability to “bounce back”) whereas the other identified resilience scales reflect the availability of assets and resources that facilitate resilience (25). Although we could not find a previous study comparing levels of resilience in stroke survivors and controls, this result does align with previous work showing reduced resilience scores in survivors of brain injury (35, 36).

We observed that higher levels of stress and lower levels of resilience were associated with higher levels of functional impairments, as indexed via the stroke impact scale (SIS). The Stroke Impact Scale (SIS) is a self-report multidimensional scale that considers strength, mobility, hand function, cognition, emotion, communication, participation, and activities of daily living, as well as a composite perceived overall recovery score. The SIS is an extensively validated instrument in the context of stroke with well-described psychometric properties, allowing the capture of multiple aspects of health and quality of life (28). A previous study measuring the factor structure of the SIS identified that strength, hand function, mobility, and activities of daily living all clustered in a unique factor referred to as “physical,” with the remaining outcomes clustering on cognitive, emotional, and social participation factors (29).

We used multiple linear regression to examine the relationship between perceived stress (PSS-10) and stroke outcomes. This analysis indicated that a higher level of perceived stress was significantly associated with worse outcomes in the SIS domains of memory, thinking, mood, emotion, participation, and role function. Interestingly, these are all cognitive and psychological/emotional outcomes. This result aligns with a small number of previous studies investigating stress levels in stroke survivors (5, 7, 8, 10). These have indicated that stress levels in stroke survivors are elevated, compared with non-stroke controls, and that stress is associated with worse outcomes (5, 7, 8, 10, 11). While causality cannot be inferred from our cross-sectional data, there are a number of potential causes of this relationship. For instance, the experience of stroke may cause heightened stress reactivity, or people with worse cognitive outcomes may experience higher levels of stress as a result. The nature of this relationship should continue to be investigated in future work.

We investigated the relationship between resilience and stroke outcome in stroke survivors using two complementary scales, the Brief Resilience Scale (BRS) and the Connor Davidson Resilience Scale (CD-RISC), thought to capture different aspects of resilience. In adjusted single predictor regression models, resilience as measured by the BRS was associated with cognitive and psychological stroke outcomes, with a higher level of resilience associated with better stroke outcomes; specifically mood and emotion, communication, participation and role function, and the overall perception of recovery. However, the regression models using the CD-RISC were not statistically significant. The separate measures of resilience were differentially associated with stroke outcomes, which may suggest that the ability to bounce back after trauma or adversity, more than the process of adaptation to adversity, is associated with stroke outcomes. This suggests that the personal qualities that feed into resilience may also be associated with the recovery trajectory after stroke.

This study adds to the growing literature around resilience in stroke outcomes, which suggests that resilience is an important contributor to stroke recovery (19, 37, 38). Previous early work showed that stroke outcomes depend on internal buffers, such as overall outlook on life, attitudes regarding the stroke, and ability to cope (39). If it is shown that increased resilience predicts better long-term stroke outcomes, the development of interventions to increase resilience, which is a modifiable construct, may therefore improve cognitive, and emotional outcomes post stroke (18, 19, 40).

Interestingly, neither stress nor resilience was associated with physical outcomes on the Stroke Impact Scale. This suggests that the relationship between physical function and psychosocial factors is less clear than for cognitive and psychological function. However, the level of perceived stress and resilience were both associated with participation/role function and the overall perception of recovery, suggesting that these factors may potentially moderate the relationship between physical outcomes and perception of overall quality of life. Collectively, our findings suggest that the psychosocial constructs of perceived stress and resilience are important correlates of long-term psychological and cognitive outcomes following stroke. This is important, as at present these outcomes occur at significantly greater rates in stroke survivors in the chronic phase of stroke compared with population averages. There are a number of promising interventions to target resilience, such as controlled breathing, grounding, and relation skills training, which may be adapted to increase resilience and therefore promote improved recovery in people who have had a stroke (12, 41–43). The findings from this study suggest that research examining this possibility is warranted.

Some limitations should be considered when interpreting these results. This study was cross-sectional and as such precludes conclusions and inferences about the causal and directional relationships among variables. There was also no external verification of the stroke diagnosis, which was self-reported in this study, and severity of stroke could therefore not be included in regression models. Perceived stress and resilience are dynamic processes and a single-point measurement of these factors may not be truly representative of the patterns of disease-related coping and resilience processes. Our cohort had a relatively high proportion of haemorrhagic stroke survivors (~37%). Although we adjusted for stroke type in the linear regression models used to analyse the results, it is unclear what impact this distribution may have had on the study results. Some studies have suggested that haemorrhagic stroke is associated with poorer long-term neurologic outcomes (44). Further, given the heterogeneity of the stroke survivor cohort and the variability in the length of time post-stroke, there may be additional stressful events beyond the impact of the stroke itself that could be contributing to the higher levels of perceived stress in this population. Therefore, the longitudinal relationship between stress, resilience, and stroke outcomes should be an important consideration in future studies.

## Conclusions

These findings add to the literature on psychological factors that may be associated with poor cognitive outcomes in the chronic phase of stroke. Both stress and resilience were independently associated with the impact of stroke in a number of cognitive domains, including mood and emotion, communication, memory and thinking, participation and role function, and overall perception of recovery. Stroke is a life-changing event that requires significant adaptation and increases vulnerability to long-term functional decline. Whilst stress is likely to be a common and natural response to stroke, it has the potential to negatively interfere with rehabilitation and recovery. This is significant as cognitive impairment is frequently associated with poorer quality of life and increased likelihood of depressive symptoms in stroke survivors (17, 45, 46). Historically, the primary focus of stroke rehabilitation research has been on physical function, with the rehabilitation of psychological and cognitive impairments receiving comparatively less attention (47, 48). Identifying factors which may modify the risk of poor cognitive function is therefore of importance for improving these outcomes. Given that cognitive and emotional outcomes of stroke in particular are not always predicted by stroke characteristics, the role of other contributing factors should be examined. The results of this study, along with other studies in the field, suggest that the relationships between stress, resilience, and stroke outcome warrant further exploration. Resilience may provide an opportunity to ameliorate the effects of stress on cognitive outcomes post-stroke, as greater levels of resilience were associated with improved cognitive outcomes post-stroke.

## Data Availability Statement

The data that support the findings of this study are available from the corresponding author on reasonable request.

## Author Contributions

FW, LO, CE, and MN conceived and designed the study. PG, WC, LO, and MK were involved in protocol development, gaining ethical approval, patient recruitment, and data collection. PG and MH were involved in data analysis. PG, MH, and FW wrote the first draft of the manuscript. All authors reviewed and edited the manuscript and approved the final version of the manuscript.

## Conflict of Interest

The authors declare that the research was conducted in the absence of any commercial or financial relationships that could be construed as a potential conflict of interest.
