# Supplementary material for: Opposing Associations of Stress and Resilience With Functional Outcomes in Stroke Survivors in the Chronic Phase of Stroke: A Cross-Sectional Study
Source: Front Neurol. 2020 Apr 22;11:230. doi: 10.3389/fneur.2020.00230 (PMC7188983; doi:10.3389/fneur.2020.00230)
Supplement: Supplementary file 1 [file Data_Sheet_1.docx]

**Supplementary data: Unadjusted (crude) linear regression models**

**Supplementary Table 1- unadjusted regression models for Table 2: Comparison of stress and resilience measures between controls and stroke survivors**

|  | **Controls, mean (SE)** | **n** | **Stroke survivors, mean (SE)** | **n** | **Unadjusted B (95% CI)** | **p-value** |
| --- | --- | --- | --- | --- | --- | --- |
| PSS-10 | 11.43 (0.7) | 70 | 16.90 (0.8) | 70 | 5.47 (3.33, 7.61) | <0.001 |
| Serum cortisol (µg/dL) | 9.3 (0.4) | 70 | 7.8 (0.4) | 68 | -1.52 (-2.67, -0.37) | 0.01 |
| Copeptin (pg/mL) | 174.6 (11.9) | 70 | 163.8 (14.1) | 68 | -10.77 (-47.12, 25.59) | 0.559 |
| BRS | 4.0 (0.1) | 69 | 3.5 (0.1) | 70 | -0.48 (-0.77, -0.19) | 0.001 |
| CD-RISC | NR | - | 69.1 (2.2) | 66 | - | - |
| Cort 1^st^ segment (pg/mg) | 14.4 (1.8) | 59 | 14.8 (2.6) | 60 | 0.46 (-5.83, 6.75) | 0.885 |
| Cort 2^nd^ segment (pg/mg) | 16.2 (2.2) | 55 | 14.4 (2.4) | 50 | 1.79 (-4.63, 8.22) | 0.581 |

Abbreviations: BRS, brief resilience Scale; CD-RISC, Connor-Davidson Resilience Scale; CI, confidence interval; PSS-10, 10 item Perceived Stress Scale; SE, standard error mean; NR,not recorded

**Supplementary Table 2- unadjusted regression models for Table 4: Main effect of perceived stress (PSS-10) on Stroke Impact Scale (SIS)**

| **Outcome** | **Exposure** | **Unstandardised coefficients** | | **Standardised coefficients** | **T** | ***p*** | **R^2^** | **F** |
| --- | --- | --- | --- | --- | --- | --- | --- | --- |
|  |  | **Beta (95% CI)** | **SE** | **Beta** |  |  |  |  |
| **Physical problems** |  |  |  |  |  |  | 0.66 | 4.774 |
|  | Intercept | 65.61 (58.94, 72.27) | 3.34 |  | 19.64 | <0.001 |  |  |
|  | PSS-10 | -0.97 (-1.86, -0.08) | 0.44 | -0.26 | -2.19 | 0.032 |  |  |
| **Memory and thinking** | |  |  |  |  |  | 0.211 | 18.13 |
|  | Intercept | 75.12 (70.24, 79.99) | 2.44 |  | 30.75 | <0.001 |  |  |
|  | PSS-10* | -1.38 (-2.03, -0.74) | 0.33 | -0.46 | -4.26 | <0.001 |  |  |
| **Mood and emotion** |  |  |  |  |  |  | 0.336 | 34.47 |
|  | Intercept | 77.63 (74.07, 81.19) | 1.79 |  | 43.47 | <0.001 |  |  |
|  | PSS-10* | -1.39 (-1.87, -0.92) | 0.24 | -0.58 | -5.87 | <0.001 |  |  |
| **Communication** |  |  |  |  |  |  | 0.164 | 13.37 |
|  | Intercept | 82.02 (77.31, 86.72) | 2.36 |  | 34.80 | <0.001 |  |  |
|  | PSS-10* | -1.15 (-1.77, -0.52) | 0.31 | -0.41 | -3.66 | <0.001 |  |  |
| **Activities of daily living** | |  |  |  |  |  | 0.111 | 8.451 |
|  | Intercept | 82.78 (77.67, 87.89) | 2.56 |  | 32.33 | <0.001 |  |  |
|  | PSS-10 | -0.99 (-1.67, -0.31) | 0.34 | -0.33 | -2.91 | 0.01 |  |  |
| **Mobility** |  |  |  |  |  |  | 0.088 | 6.536 |
|  | Intercept | 80.53 (75.40, 85.65) | 2.57 |  | 31.34 | <0.001 |  |  |
|  | PSS-10 | - 0.87 (-1.56, -0.19) | 0.34 | -0.30 | -2.56 | 0.01 |  |  |
| **Hand function** |  |  |  |  |  |  | 0.039 | 2.732 |
|  | Intercept | 62.85 (53.65, 72.04) | 4.61 |  | 13.64 | <0.001 |  |  |
|  | PSS-10 | -1.01 (-2.24, 0.21) | 0.61 | -0.20 | -1.65 | 0.10 |  |  |
| **Participation/ role function** | |  |  |  |  |  | 0.216 | 18.79 |
|  | Intercept | 68.20 (62.74, 73.66) | 2.74 |  | 24.93 | <0.001 |  |  |
|  | PSS-10* | -1.58 (-2.30, -0.85) | 0.36 | -0.47 | -4.33 | <0.001 |  |  |
| **Overall perception of recovery** | |  |  |  |  |  | 0.159 | 12.87 |
|  | Intercept | 70.68 (66.24, 75.12) | 2.23 |  | 31.75 | <0.001 |  |  |
|  | PSS-10* | -1.06 (-1.65, -0.47) | 0.30 | -0.40 | -3.59 | 0.001 |  |  |

Notes: PSS-10 mean centred. Threshold p-value 0.00185 to account for multiple comparisons

^*^Statistically significant

**Supplementary Table 3- unadjusted regression models for Table 5: Main effect of resilience (BRS) on Stroke Impact Scale (SIS)**

| **Outcome** | **Exposure** | **Unstandardised coefficients** | | **Standardised coefficients** | **T** | ***p*** | **R^2^** | **F** |
| --- | --- | --- | --- | --- | --- | --- | --- | --- |
|  |  | **Beta (95% CI)** | **SE** | **Beta** |  |  |  |  |
| **Physical problems** | |  |  |  |  |  | 0.078 | 5.765 |
|  | Intercept | 64.93 58.55 71.31 | 3.20 |  | 20.30 | <0.001 |  |  |
|  | BRS | 8.33 1.41 15.26 | 3.47 | 0.28 | 2.40 | 0.019 |  |  |
| **Memory and thinking** | |  |  |  |  |  | 0.161 | 13.076 |
|  | Intercept | 73.59 68.75 78.44 | 2.43 |  | 30.32 | <0.001 |  |  |
|  | BRS | 9.52 4.27 14.77 | 2.63 | 0.40 | 3.62 | 0.001 |  |  |
| **Mood and emotion** | |  |  |  |  |  | 0.261 | 24.042 |
|  | Intercept | 76.11 72.49 79.73 | 1.82 |  | 41.90 | <0.001 |  |  |
|  | BRS | 9.66 5.73 13.59 | 1.97 | 0.51 | 4.90 | <0.001 |  |  |
| **Communication** |  |  |  |  |  |  | 0.288 | 27.446 |
|  | Intercept | 81.72 77.53 85.90) | 2.10 |  | 38.95 | <0.001 |  |  |
|  | BRS | 11.92 7.38 16.46 | 2.28 | 0.54 | 5.24 | <0.001 |  |  |
| **Activities of daily living** | |  |  |  |  |  | 0.067 | 4.905 |
|  | Intercept | 81.52 76.47 86.56 | 2.53 |  | 32.25 | <0.001 |  |  |
|  | BRS | 6.07 0.60 11.54 | 2.74 | 0.26 | 2.22 | 0.03 |  |  |
| **Mobility** |  |  |  |  |  |  | 0.023 | 1.565 |
|  | Intercept | 78.96 73.85 84.08 | 2.56 |  | 30.80 | <0.001 |  |  |
|  | BRS | 3.48 -2.07 9.03 | 2.78 | 0.15 | 1.25 | 0.215 |  |  |
| **Hand function** |  |  |  |  |  |  | 0.016 | 1.115 |
|  | Intercept | 61.30 52.33 70.27 | 4.50 |  | 13.64 | <0.001 |  |  |
|  | BRS | 5.15 -4.58 14.88 | 4.88 | 0.13 | 1.06 | 0.295 |  |  |
| **Participation/ role function** | |  |  |  |  |  | 0.197 | 16.668 |
|  | Intercept | 66.70 61.37 72.03 | 2.67 |  | 24.98 | <0.001 |  |  |
|  | BRS* | 11.82 6.05 17.60 | 2.90 | 0.44 | 4.08 | <0.001 |  |  |
| **Overall perception of recovery** | |  |  |  |  |  | 0.208 | 17.812 |
|  | Intercept | 70.04 (65.89, 74.20) | 2.08 |  | 33.62 | <0.001 |  |  |
|  | BRS* | 9.54 (5.03, 14.05) | 2.26 | 0.46 | 4.22 | <0.001 |  |  |

Notes: BRS mean centred. Threshold p-value 0.00185 to account for multiple comparisons

^*^Statistically significant

**Supplementary Table 4- unadjusted regression models for Table 6: Main effect of resilience (CD-RISC) on Stroke Impact Scale (SIS)**

| **Outcome** | **Exposure** | **Unstandardised coefficients** | | **Standardised coefficients** | **T** | ***p*** | **R^2^** | **F** |
| --- | --- | --- | --- | --- | --- | --- | --- | --- |
|  |  | **Beta (95% CI)** | **SE** | **Beta** |  |  |  |  |
| **Physical problems** | |  |  |  |  |  | 0.027 | 1.758 |
|  | Intercept | 62.50 55.82 69.18 | 3.34 |  | 18.69 | <0.001 |  |  |
|  | CD-RISC | 0.25 -0.13 0.62 | 0.19 | 0.16 | 1.33 | 0.190 |  |  |
| **Memory and thinking** | |  |  |  |  |  | 0.137 | 10.185 |
|  | Intercept | 71.16 66.25 76.07 | 2.46 |  | 28.94 | <0.001 |  |  |
|  | CD-RISC | 0.44 0.16 0.72 | 0.14 | 0.37 | 3.19 | 0.002 |  |  |
| **Mood and emotion** | |  |  |  |  |  | 0.186 | 14.649 |
|  | Intercept | 72.94 69.14 76.74 | 1.90 |  | 38.31 | <0.001 |  |  |
|  | CD-RISC* | 0.41 0.20 0.62 | 0.11 | 0.43 | 3.83 | <0.001 |  |  |
| **Communication** |  |  |  |  |  |  | 0.102 | 7.234 |
|  | Intercept | 78.19 73.44 82.95 | 2.38 |  | 32.84 | <0.001 |  |  |
|  | CD-RISC | 0.36 0.09 0.63 | 0.13 | 0.32 | 2.69 | 0.009 |  |  |
| **Activities of daily living** | |  |  |  |  |  | 0.057 | 3.884 |
|  | Intercept | 80.15 75.18 85.13 | 2.49 |  | 32.19 | <0.001 |  |  |
|  | CD-RISC | 0.28 0.00 0.55 | 0.14 | 0.24 | 1.97 | 0.053 |  |  |
| **Mobility** |  |  |  |  |  |  | 0.025 | 1.612 |
|  | Intercept | 78.58 73.62 83.54 | 2.48 |  | 31.67 | <0.001 |  |  |
|  | CD-RISC | 0.18 -0.10 0.45 | 0.14 | 0.16 | 1.27 | 0.209 |  |  |
| **Hand function** |  |  |  |  |  |  | 0.008 | 0.484 |
|  | Intercept | 59.70 50.61 68.79 | 4.55 |  | 13.12 | <0.001 |  |  |
|  | CD-RISC | 0.18 -0.33 0.69 | 0.26 | 0.09 | 0.70 | 0.489 |  |  |
| **Participation/ role function** | |  |  |  |  |  | 0.116 | 8.365 |
|  | Intercept | 63.02 57.39 68.66 | 2.82 |  | 22.33 | <0.001 |  |  |
|  | CD-RISC | 0.46 0.14 0.77 | 0.16 | 0.34 | 2.89 | 0.005 |  |  |
| **Overall perception of recovery** | |  |  |  |  |  | 0.113 | 8.19 |
|  | Intercept | 67.87 (63.41, 72.32) | 2.23 |  | 30.46 | <0.001 |  |  |
|  | CD-RISC | 0.36 (0.12, 0.61) | 0.13 | 0.34 | 2.86 | 0.006 |  |  |

Notes: CD-RISC mean centred. Threshold p-value 0.00185 to account for multiple comparisons

^*^Statistically significant
