# Supplementary material for: Opposing Associations of Stress and Resilience With Functional Outcomes in Stroke Survivors in the Chronic Phase of Stroke: A Cross-Sectional Study
Source: Front Neurol. 2020 Apr 22;11:230. doi: 10.3389/fneur.2020.00230 (PMC7188983; doi:10.3389/fneur.2020.00230)
Supplement: Supplementary file 2 [file Data_Sheet_2.docx]

**STROBE (Strengthening the Reporting of Observational Studies in Epidemiology) Statement** **reporting checklist for cross-sectional study**

| **Section and topic** | Item No | Recommendation and location in document |
| --- | --- | --- |
| **Title and abstract** |  |  |
| Title | 1a | Indicate the study’s design with a commonly used term in the title or the abstract – *In both the title and the abstract this is explicitly described as a cross-sectional study.* |
| Abstract | 1b | Provide in the abstract an informative and balanced summary of what was done and what was found – *Stated in abstract.* |
| Introduction | | |
| Background/rationale | 2 | Explain the scientific background and rationale for the investigation being reported – *Stated in the Introduction* |
| Objectives | 3 | State specific objectives, including any prespecified hypotheses – *Aims and hypotheses stated* |
| Methods | | |
| Study design | 4 | Present key elements of study design early in the paper – *Stated at the start of Methods* |
| Setting | 5 | Describe the setting, locations, and relevant dates, including periods of recruitment, exposure, follow-up, and data collection – *Stated in Methods* |
| Eligibility criteria | 6a | Give the eligibility criteria, and the sources and methods of selection of participants. Describe methods of follow-up – *eligibility criteria, sources and methods of selection* *were stated in Methods; no follow-up (cross-sectional study at a single time point)* |
|  | 7 | Clearly define all outcomes, exposures, predictors, potential confounders, and effect modifiers – *Clearly defined in Methods*  Give diagnostic criteria, if applicable – *NA* |
| Data sources/ measurement | 8 | For each variable of interest give sources of data and details of methods of assessment (measurement). Describe comparability of assessment methods if there is more than one group. Give information separately for exposed and unexposed groups if applicable– *Described in Methods*. *References for all measures provided* |
| Bias | 9 | Describe any efforts to address potential sources of bias – *Sensitivity analysis conducted and limitations acknowledged* |
| Study size | 10 | Explain how the study size was arrived at – *Stated in Methods (sample size calculation)* |
| Quantitative variables | 11 | Explain how quantitative variables were handled in the analyses. If applicable, describe which groupings were chosen and why – *Stated in Methods (Measures and Statistical Analysis)* |
| Statistical methods | 12a | Describe all statistical methods, including those used to control for confounding – *Stated in Methods (Statistical Analysis)* |
|  | 12b | Describe any methods used to examine subgroups and interactions – *NA* |
|  | 12c | Explain how missing data were addressed – *Defined in Methods (Statistical analysis)* |
|  | 12d | If applicable, describe analytical methods taking account of sampling strategy – *NA* |
|  | 12e | Describe any sensitivity analyses – *Sensitivity analysis conducted as stated in Methods and Results* |
| Results | | |
| Participants | 13a | Report numbers of individuals at each stage of study—eg numbers potentially eligible, examined for eligibility, confirmed eligible, included in the study, completing follow-up, and analysed – *Presented in Methods and Results (Table 1)* |
|  | 13b | Give reasons for non-participation at each stage – *NA* |
|  | 13c | Consider use of a flow diagram – *NA* |
| Descriptive data | 14a | Give characteristics of study participants (eg demographic, clinical, social) and information on exposures and potential confounders – *Stated in Results (Descriptive Statistics)* |
|  | 14b | Indicate number of participants with missing data for each variable of interest – *Indicated in Tables 1 and 2* |
| Outcome data | 15 | Report numbers of outcome events or summary measures. Give information separately for exposed and unexposed groups if applicable – *NA* |
| Main results | 16a | Give unadjusted estimates and, if applicable, confounder-adjusted estimates and their precision (eg, 95% confidence interval). Make clear which confounders were adjusted for and why they were included – *Indicated in Methods and Results* |
|  | 16b | Report category boundaries when continuous variables were categorized – *NA* |
|  | 16c | If relevant, consider translating estimates of relative risk into absolute risk for a meaningful time period – *NA* |
| Other analyses | 17 | Report other analyses done—eg analyses of subgroups and interactions, and sensitivity analyses – *Sensitivity analysis presented in Results* |
| Discussion | | |
| Key results | 18 | Summarise key results with reference to study objectives – *Addressed in Discussion* |
| Limitations | 19 | Discuss limitations of the study, taking into account sources of potential bias or imprecision. Discuss both direction and magnitude of any potential bias – *Described in Discussion* |
| Interpretation | 20 | Give a cautious overall interpretation of results considering objectives, limitations, multiplicity of analyses, results from similar studies, and other relevant evidence – *Cautious interpretation provided in Discussion* |
| Generalisability | 21 | Discuss the generalisability (external validity) of the study results – *Addressed in Discussion* |
| Other information | | |
| Funding | 22 | Give the source of funding and the role of the funders for the present study and, if applicable, for the original study on which the present article is based – *Funding sources presented.* |

**Note:** An explanation and elaboration article discusses each checklist item and gives methodological background and published examples of transparent reporting. The STROBE checklist is best used in conjunction with this article (freely available on the websites of PLoS Medicine at http://www.plosmedicine.org/, Annals of Internal Medicine at http://www.annals.org/, and Epidemiology at http://www.epidem.com/). Information on the STROBE Initiative is available at http://www.strobe-statement.org.
